# Gene expression profiling of peripheral blood mononuclear cells in the setting of peripheral arterial disease

**DOI:** 10.1186/2043-9113-2-6

**Published:** 2012-03-12

**Authors:** Rizwan Masud, Khader Shameer, Aparna Dhar, Keyue Ding, Iftikhar J Kullo

**Affiliations:** 1 Division of Cardiovascular Diseases, Mayo Clinic, Rochester MN 55905, USA

**Keywords:** Peripheral arterial disease, Gene expression, Microarray analysis, Vascular disease, Biomarkers

## Abstract

**Background:**

Peripheral arterial disease (PAD) is a relatively common manifestation of systemic atherosclerosis that leads to progressive narrowing of the lumen of leg arteries. Circulating monocytes are in contact with the arterial wall and can serve as reporters of vascular pathology in the setting of PAD. We performed gene expression analysis of peripheral blood mononuclear cells (PBMC) in patients with PAD and controls without PAD to identify differentially regulated genes.

**Methods:**

PAD was defined as an ankle brachial index (ABI) ≤0.9 (n = 19) while age and gender matched controls had an ABI > 1.0 (n = 18). Microarray analysis was performed using Affymetrix HG-U133 plus 2.0 gene chips and analyzed using GeneSpring GX 11.0. Gene expression data was normalized using Robust Multichip Analysis (RMA) normalization method, differential expression was defined as a fold change ≥1.5, followed by unpaired Mann-Whitney test (*P < 0.05*) and correction for multiple testing by Benjamini and Hochberg False Discovery Rate. Meta-analysis of differentially expressed genes was performed using an integrated bioinformatics pipeline with tools for enrichment analysis using Gene Ontology (GO) terms, pathway analysis using Kyoto Encyclopedia of Genes and Genomes (KEGG), molecular event enrichment using Reactome annotations and network analysis using Ingenuity Pathway Analysis suite. Extensive biocuration was also performed to understand the functional context of genes.

**Results:**

We identified 87 genes differentially expressed in the setting of PAD; 40 genes were upregulated and 47 genes were downregulated. We employed an integrated bioinformatics pipeline coupled with literature curation to characterize the functional coherence of differentially regulated genes.

**Conclusion:**

Notably, upregulated genes mediate immune response, inflammation, apoptosis, stress response, phosphorylation, hemostasis, platelet activation and platelet aggregation. Downregulated genes included several genes from the zinc finger family that are involved in transcriptional regulation. These results provide insights into molecular mechanisms relevant to the pathophysiology of PAD.

## Introduction

Peripheral arterial disease (PAD) affects more than eight million adults in the United States and is associated with significant mortality and morbidity [1-6]. PAD is a surrogate for diffuse atherosclerosis but is often underdiagnosed [4,6]. Identification of differentially regulated genes in the setting of PAD may lead to potential biomarkers for the earlier detection and prognostication of this disease and provide insights into its pathophysiology.

Gene expression analysis of peripheral blood mononuclear cells (PBMC) in asymptomatic individuals has previously revealed individual genetic variation and differentially regulated expression patterns [7,8]. Circulating peripheral blood cells have been used to examine differentially regulated genes in several cardiovascular disorders. For example, gene expression profiling studies of blood cells have identified differentially regulated genes and pathways in hypertension [9], coronary artery disease [10,11] and ischemic stroke [1,10,12-16]. However, genes differentially expressed in PBMC in the setting of PAD have yet to be identified. Circulating PBMC are in contact with the arterial wall and may be useful in investigating molecular mechanisms relevant to PAD. We therefore performed gene expression analysis to identify differentially expressed genes in PBMC in the setting of PAD.

## Materials and methods

### Participant recruitment and sample characteristics

The Mayo Clinic Institutional Review Board approved the study and all participants provided written informed consent. The participants were recruited from the Mayo non-invasive vascular laboratory and PAD was defined as an ankle brachial index (ABI) ≤0.9 (n = 19) while age and gender matched controls had an ABI > 1.0 (n = 18). ABI was measured in both the lower extremities and the lower of the two values was recorded for the analysis [17]. Individuals with poorly compressible arteries or aortic aneurysmal disease were excluded.

### Isolation of peripheral blood mononuclear cells (PBMC) and RNA isolation

PBMC were isolated by density gradient centrifugation by layering the blood samples over histopaque (Sigma-Aldrich, St. Louis, MO),[18]. In brief, 18 ml of whole blood was mixed with equal amount of PBS (Bio-Rad, Hercules, CA), and layered over 12 ml of histopaque 1077 (used for cell separation). The PBMC layer was removed, washed, and centrifuged twice with Hank's Balanced Salt Solution (HBSS) (Sigma-Aldrich, St. Louis, MO). The pellet formed after double centrifugation was re-suspended in Complete RPMI-10 medium. The cells were counted using a hemocytometer and processed for RNA isolation using RNeasy Plus Mini Kit (Qiagen, Valencia, CA), and additionally with TRIzol (Invitrogen, Carlsbad, CA). For the RNeasy kit, PBMCs were disrupted and homogenized using RLT buffer (Qiagen, Valencia, CA). The RNeasy kit includes gDNA eliminator spin column for the removal of genomic DNA from the sample, allowing subsequent purification of RNA. The flow through from the gDNA column was mixed with ethanol and placed on the RNeasy spin column. This spin column uses a silica gel based membrane for effective binding and purification of RNA. Total RNA was eluted in RNase free water, quantified using NanoDrop 1000 (Thermo Scientific, Wilmington, DE) and stored at −80°C.

### Microarray analysis

RNA quantity and quality were assessed using Agilent 2100 Bioanalyzer (Agilent, Santa Clara, CA); 100 ng of total RNA was used for generation of biotin labeled cRNA using Affymetrix Two-Cycle cDNA Synthesis Kit (Affymetrix, Santa Clara, CA). After the first cycle, in vitro transcription-based (IVT) amplification of cRNA was carried out using MEGAscript T7 kit (Applied Biosystems/Ambion, Austin TX). With the second cycle cDNA synthesis, biotin labeled cRNA was generated using the Affymetrix IVT labeling Kit. The labeled cRNA was cleaned, quantified and after fragmentation, hybridized to Affymetrix HG-U133 Plus2.0 GeneChips. The chips were stained with streptavidin phycoerythrin and biotinylated antibody and washed at an Affymetrix Fluidics station 450. The GeneChips were scanned and data extracted using GeneChip scanner 3000 (Affymetrix, Santa Clara, CA) and the raw data file formats were generated using GeneChip operating software (GCOS).

### Data processing and statistical analysis

**Figure 1:**
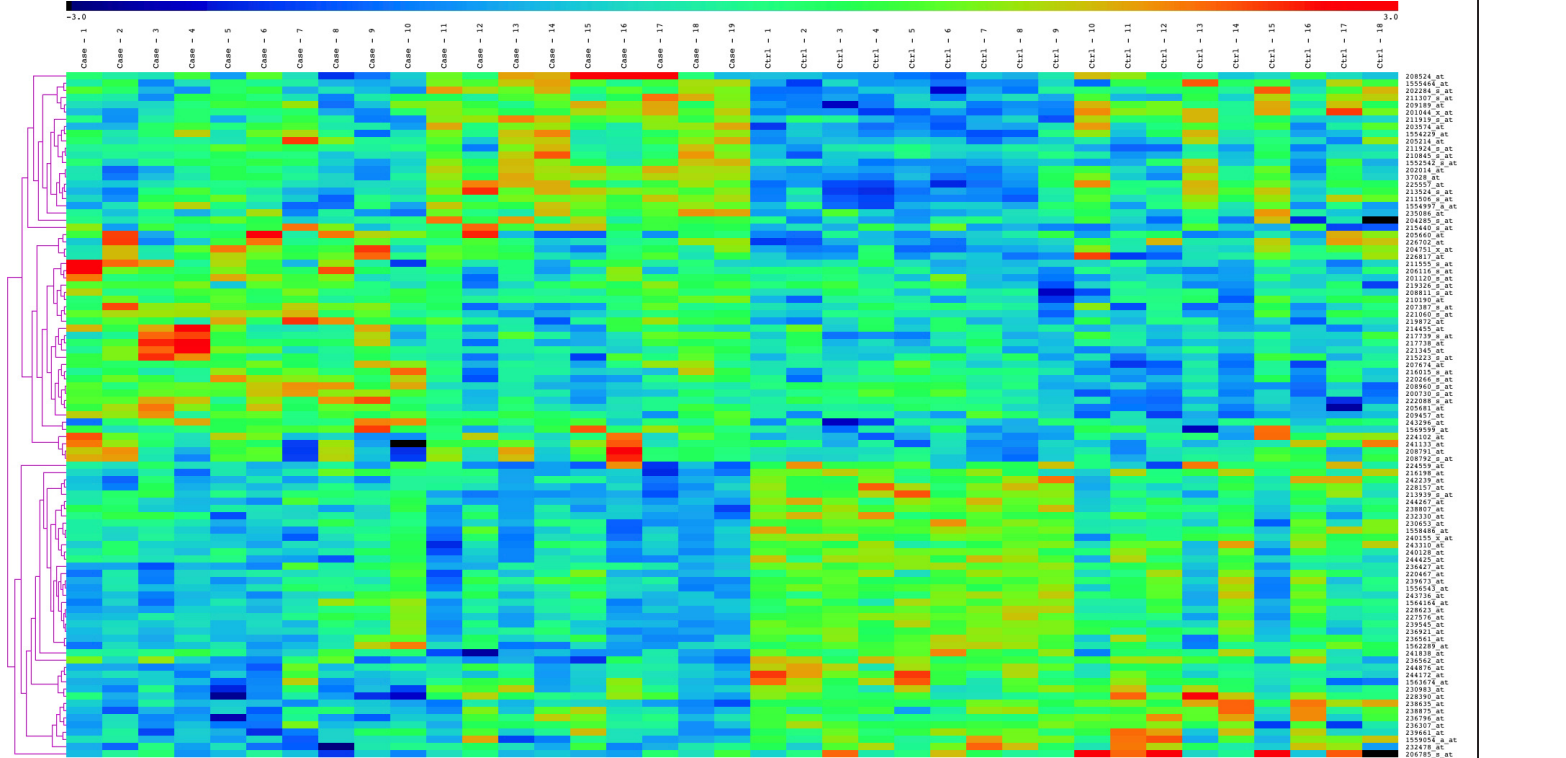
**Hierarchical clustering of differentially regulated genes in the setting of PAD**. Clustering was performed using hierarchical clustering algorithm with Euclidean distance and average linkage clustering method.

Raw gene expression data were analyzed using the GeneSpringGx 11.0 software (Agilent^® ^Technologies, Santa Clara, CA). All samples were normalized and summarized by Robust Multichip Analysis (RMA) normalization method, which includes background correction, normalization and calculation of expression values [19]. Baseline was set to median for all samples, where median of the log-transformed value of each probe from all samples was calculated and this value was subtracted from all samples. Probes were filtered and eliminated on expression level as part of quality control (QC) and probes with expression values <20% were excluded. Of the remaining probes, those with a 1.5-fold-change difference between the groups underwent unpaired Mann-Whitney test and multiple testing correction was performed using Benjamini and Hochberg False Discovery Rate (FDR). Following statistical analysis and probe mapping, 47 genes were upregulated and 39 genes were downregulated. Differentially expressed probes were clustered using MultiExperiment Viewer (MeV v4.5 [20]) (Figure 1). The clustering figure shows a distinct pattern of upregulated and downregulated probes in cases when compared to the controls.

### Validation using real-time PCR

To validate our findings from gene expression analysis, we performed real-time PCR of the Syntaxin 11 (*STX11)*, a gene not previously associated with PAD. *STX11 *is a component of t-SNARE complex and involved in endocytic vesicular transport, regulate protein transport among late endosomes and the trans-Golgi network and may have functional or regulatory role in vascular diseases. RNA derived from PAD and control samples was converted to cDNA by reverse transcription using Transcriptor First Strand cDNA Synthesis kit (Roche, OH, USA) and used immediately for real-time PCR. The FASTA sequence of each gene of interest was obtained using NCBI nucleotide search to design the primers http://www.ncbi.nlm.nih.gov/nuccore. This FASTA sequence was used as query to search in NCBI Primer BLAST http://www.ncbi.nlm.nih.gov/tools/primer-blast/index.cgi?LINK_LOC=BlastHome. Primers pairs were selected based on primer length (18-30 bp), GC content, melting temperature (T_m _= 59-60°C), and product size. Primers were selected that scan exon-exon junctions. BLAST was used to check the specificity of primers to the gene of interest. PAGE-purified oligos (Integrated DNA Technologies, IA, USA) were used for real-time PCR. Primer sequences are available from the corresponding author upon request. Real-time PCR assay was performed using the LightCycler 480 instrument (Roche) and the LightCycler 480 SYBR Green I Master kit and protocol. Each sample was assayed in duplicate for the genes of interest as well as ß-actin (*ACTB*) as a housekeeping gene for normalization. Samples were assayed in 384-well plates with a 20 μL reaction volume. (10 uL master mix (FastStart Taq DNA polymerase, reaction buffer, dNTPs, SYBR Green I dye, and MgCl_2_), 3uL PCR-grade water, 1 uL each 2 nM primer, and 5 uL (32 ng) cDNA template. Raw Cp values were calculated using the Abs Quant/2^nd ^derivative max option in Roche's LightCycler 480 software (release 1.5.0 SP3).

### Functional annotation of differentially regulated genes using *in-silico *approach

To assess the functional repertoire of differentially expressed genes we adopted a multi-tiered bioinformatics annotation pipeline with functional enrichment calculations, pathway and molecular event analysis, biological network analysis and biocuration. Statistically significant genes and annotations were used as pointers to perform literature curation to derive biological role of genes differentially regulated in the setting of PAD. Preliminary functional annotations of differentially regulated genes were derived using BioGPS [21].

GO term enrichment analysis was performed using DAVID v6.7 [22,23]. As no single annotation resource provides information about all available biological pathways, we used two different pathway databases (Reactome v36.2 [24] and Kyoto Encyclopedia of Genes and Genomes (KEGG) [25]) to identify the biological pathways mediated by differentially expressed genes. Reactome based pathway enrichment analysis was performed using Reactome Pathway Analysis tool http://www.reactome.org/ReactomeGWT/entrypoint.html#PathwayAnalysisDataUploadPage. This two-fold approach was useful in finding several relevant pathways from two different pathway analyses. Ingenuity Pathway Analysis^® ^suite (IPA v9.0 - 3211, http://www.ingenuity.com) was used to understand functional networks involved in the gene sets. IPA-Tox^®^, a data analysis routine within IPA that assess potential toxicity with various compounds using toxicogenomics data was also used to interpret the functional context of differentially regulated genes. Finally, to understand the functional context and biological significance of differentially expressed genes relevant to PAD, scientific literature was curated using Gene Related InFormation (GeneRIF http://www.ncbi.nlm.nih.gov/projects/GeneRIF) and 'related articles by PubMed' in the 'Entrez Gene' http://www.ncbi.nlm.nih.gov/gene page.

## Results

Patient characteristics are summarized in Table 1. The PBMC from patients with PAD differentially expressed 87 genes involved in immune response, inflammation, phosphorylation, signal transduction, platelet aggregation, vitamin metabolism, hemostasis, oxidative stress and transcriptional regulation.

**Table 1:** Sample characteristics

Characteristic	Samples		*P-value*
	
	Cases (n = 19)	Controls (n = 18)	
Age, years	69.57 ± 9.18	66.88 ± 9.44	0.54

Men, %	13 (68%)	13 (72%)	0.99

Ever smoker, %	17 (89%)	12 (66%)	0.15

BMI, kg⋅m^-2^	28.53 ± 5.25	30.6 ± 5.99	0.27

Systolic BP, mm Hg	139.2 ± 23.27	132.4 ± 20.1	0.47

Diastolic BP, mm Hg	74.2 ± 11.66	80.1 ± 12.3	0.15

HDL cholesterol, mg/dl	44.12 ± 8.59	49.7 ± 15	0.38

Hypertension, %	17 (89.4%)	13 (72%)	0.18

Diabetes, %	5 (26%)	4 (22%)	0.69

Lipid-lowering medication, %	11 (57%)	12(66%)	0.85

Ankle-brachial index	0.48 ± 0.20	1.12 ± 0.07	<0.001

WBC count, μL	8.03 ± 2.34	7.26 ± 1.10	0.18

Values are expressed as either 'mean ± standard deviation' or as 'n (%)'

### Differentially expressed genes

Based on the comparisons of cases and controls, after initial data filtering and at a 1.5 fold change, 95 probes were differentially expressed (*P < 0.05; *) (Table 2). A subset of probes was not annotated in Affymetrix annotation files. We obtained enhanced probe mapping by combining results from multiple probe mapping tools AILUN [26], BioMart [27] and GATExplorer [28]. Final probe mapping was performed using the union of mapped results obtained from these methods. NCBI-Gene and UCSC genome browser [29] were also consulted for annotation and integration of probe and gene related information. Statistically significant probes, mapped genes, *P-value*, fold change absolute (FCA) and directions of expression (regulation) are summarized in Table 2. Ten downregulated probes were not mapped to a valid gene using the probe mapping approach that we employed and were manually mapped using Ensembl v58 annotations [30]. For example probe "241838_at" (FCA 1.53) was not mapped to a valid gene using our probe mapping strategy but using Ensembl v58 [30] the probe was mapped to a non-coding transcript RP1-167A14.2. One probe "243310_at" could not be mapped to any known gene using the probe mapping databases or Ensembl annotations.

**Table 2:** Differentially expressed probes/genes in the setting of peripheral arterial disease

Upregulated Probe ID	Gene name (HUGO)	Gene symbol	*P-value*	FCA
219326_s_at	UDP-GlcNAc:betaGal beta-1,3-N-acetylglucosaminyltransferase 2	*B3GNT2*	0.032	1.53
205681_at	BCL2-related protein A1	*BCL2A1*	0.008	1.73
215440_s_at	brain expressed, X-linked 4	*BEX4*	0.004	1.50
1554229_at	chromosome 5 open reading frame 41	*C5orf41*	0.004	1.51
202284_s_at	cyclin-dependent kinase inhibitor 1A (p21, Cip1)	*CDKN1A*	0.024	1.62
208791_at	clusterin	*CLU*	0.029	1.84
208792_s_at	clusterin	*CLU*	0.038	1.83
226702_at	cytidine monophosphate (UMP-CMP) kinase 2, mitochondrial	*CMPK2*	0.039	1.54
225557_at	cysteine-serine-rich nuclear protein 1	*CSRNP1*	0.032	1.57
211919_s_at	chemokine (C-X-C motif) receptor 4	*CXCR4*	0.039	1.59
208811_s_at	DnaJ (Hsp40) homolog, subfamily B, member 6	*DNAJB6*	0.004	1.53
204751_x_at	desmocollin 2	*DSC2*	0.007	1.68
226817_at	desmocollin 2	*DSC2*	0.007	1.99
201044_x_at	dual specificity phosphatase 1	*DUSP1*	0.043	1.94
209457_at	dual specificity phosphatase 5	*DUSP5*	0.008	1.56
219872_at	family with sequence similarity 198, member B	*FAM198B*	0.003	1.51
207674_at	Fc fragment of IgA, receptor for	*FCAR*	0.012	1.99
211307_s_at	Fc fragment of IgA, receptor for	*FCAR*	0.012	1.72
221345_at	free fatty acid receptor 2	*FFAR2*	0.007	2.34
209189_at	FBJ murine osteosarcoma viral oncogene homolog	*FOS*	0.01	1.70
213524_s_at	G0/G1switch 2	*G0S2*	0.024	3.90
207387_s_at	glycerol kinase	*GK*	0.035	1.61
208524_at	G protein-coupled receptor 15	*GPR15*	0.024	1.66
211555_s_at	guanylate cyclase 1, soluble, beta 3	*GUCY1B3*	0.011	1.59
214455_at	histone cluster 1, H2bg	*HIST1H2BG*	0.041	1.88
1555464_at	interferon induced with helicase C domain 1	*IFIH1*	0.026	1.55
211506_s_at	interleukin 8	*IL8*	0.022	3.69
220266_s_at	Kruppel-like factor 4 (gut)	*KLF4*	0.034	1.61
208960_s_at	Kruppel-like factor 6	*KLF6*	0.038	1.88
217738_at	nicotinamide phosphoribosyltransferase	*NAMPT*	0.027	1.73
217739_s_at	nicotinamide phosphoribosyltransferase	*NAMPT*	0.021	1.80
243296_at	nicotinamide phosphoribosyltransferase	*NAMPT*	0.008	2.00
203574_at	nuclear factor, interleukin 3 regulated	*NFIL3*	0.008	1.55
216015_s_at	NLR family, pyrin domain containing 3	*NLRP3*	0.041	1.52
205660_at	2'-5'-oligoadenylate synthetase-like	*OASL*	0.035	1.50
224102_at	purinergic receptor P2Y, G-protein coupled, 12	*P2RY12*	0.022	1.56
201120_s_at	progesterone receptor membrane component 1	*PGRMC1*	0.008	1.54
210845_s_at	plasminogen activator, urokinase receptor	*PLAUR*	0.004	1.60
211924_s_at	plasminogen activator, urokinase receptor	*PLAUR*	0.004	1.76
204285_s_at	phorbol-12-myristate-13-acetate-induced protein 1	*PMAIP1*	0.005	1.52
202014_at	protein phosphatase 1, regulatory (inhibitor) subunit 15A	*PPP1R15A*	0.012	1.96
37028_at	protein phosphatase 1, regulatory (inhibitor) subunit 15A	*PPP1R15A*	0.008	1.87
1554997_a_at	prostaglandin-endoperoxide synthase 2 (prostaglandin G/H synthase and cyclooxygenase)	*PTGS2*	0.025	2.77
200730_s_at	protein tyrosine phosphatase type IVA, member 1	*PTP4A1*	0.03	1.62
1569599_at	SAM domain, SH3 domain and nuclear localization signals 1	*SAMSN1*	0.006	1.72
222088_s_at	solute carrier family 2 (facilitated glucose transporter), member 14	*SLC2A14*	0.033	1.56
215223_s_at	superoxide dismutase 2, mitochondrial	*SOD2*	0.038	1.57
205214_at	serine/threonine kinase 17b	*STK17B*	0.004	1.60
210190_at	syntaxin 11	*STX11*	0.003	1.65
1552542_s_at	T-cell activation RhoGTPase activating protein	*TAGAP*	0.004	1.66
235086_at	thrombospondin 1	*THBS1*	0.038	2.12
221060_s_at	toll-like receptor 4	*TLR4*	0.025	1.82
206116_s_at	tropomyosin 1 (alpha)	*TPM1*	0.013	1.58
241133_at	T cell receptor beta constant 1	*TRBC1*	0.048	1.91

**Downregulated Probe ID**	**Gene name (HUGO)**	**Gene name**	***P-value***	**FCA**
239661_at	AF4/FMR2 family, member 1	*AFF1*	0.022	1.63
220467_at	--	*AL590452.1*	0.043	1.56
236921_at	*--*	*AL592494.5*	0.027	1.64
238807_at	ankyrin repeat domain 46	*ANKRD46*	0.038	1.53
216198_at	activating transcription factor 7 interacting protein	*ATF7IP*	0.026	1.50
236307_at	BTB and CNC homology 1, basic leucine zipper transcription factor 2	*BACH2*	0.039	2.11
236796_at	BTB and CNC homology 1, basic leucine zipper transcription factor 2	*BACH2*	0.015	1.70
244172_at	B-cell linker	*BLNK*	0.043	1.78
227576_at	BMP2 inducible kinase-like	*BMP2KL*	0.048	1.83
244425_at	chromosome 14 open reading frame 43	*C14orf43*	0.03	1.65
238635_at	chromosome 5 open reading frame 28	*C5orf28*	0.00E+00	2.03
232330_at	chromosome 7 open reading frame 44	*C7orf44*	0.046	1.58
239545_at	CAS1 domain containing 1	*CASD1*	0.024	1.64
1564164_at	DENN/MADD domain containing 1B	*DENND1B*	0.048	1.53
230653_at	DIS3 mitotic control homolog (S. cerevisiae)-like 2	*DIS3L2*	0.039	1.77
244876_at	early B-cell factor 1	*EBF1*	0.034	1.50
230983_at	family with sequence similarity 129, member C	*FAM129C*	0.043	1.63
1563674_at	Fc receptor-like 2	*FCRL2*	0.041	1.68
228623_at	FTX transcript, XIST regulator (non-protein coding)	*FTX*	0.048	1.82
1562289_at	G protein-coupled receptor 141	*GPR141*	0.036	1.66
206785_s_at	killer cell lectin-like receptor subfamily C, member 1	*KLRC1*	0.035	1.69
224559_at	metastasis associated lung adenocarcinoma transcript 1 (non-protein coding)	*MALAT1*	0.032	1.68
243736_at	methyltransferase like 15	*METT5D1*	0.034	1.59
232478_at	MIR181A2 host gene (non-protein coding)	*MIR181A2HG*	0.021	1.52
228623_at	FTX transcript, XIST regulator (non-protein coding)	*FTX*	0.048	1.82
243310_at	--	*--*	0.017	1.52
239673_at	nuclear receptor subfamily 3, group C, member 2	*NR3C2*	0.037	1.94
240128_at	5'-nucleotidase, cytosolic III	*NT5C3*	0.03	1.57
1559054_a_at	protein phosphatase 1, regulatory (inhibitor) subunit 7	*PPP1R7*	0.004	1.60
238875_at	RANBP2-like and GRIP domain containing 1	*RGPD1*	0.026	1.50
241838_at	--	*RP1-167A14.2*	0.034	1.54
242239_at	--	*RP11-139J15.3*	0.008	1.55
228390_at	--	*RP11-659G9.3001*	0.033	1.57
213939_s_at	RUN and FYVE domain containing 3	*RUFY3*	0.035	1.50
244267_at	SATB homeobox 1	*SATB1*	0.025	1.81
236561_at	transforming growth factor, beta receptor 1	*TGFBR1*	0.033	1.66
236427_at	WW domain containing oxidoreductase	*WWOX*	0.035	1.61
1556543_at	zinc finger, CCHC domain containing 7	*ZCCHC7*	0.041	1.68
228157_at	zinc finger protein 207	*ZNF207*	0.027	2.01
236562_at	zinc finger protein 439	*ZNF439*	0.015	1.51
240155_x_at	zinc finger protein 479	*ZNF479*	0.012	1.52
1558486_at	zinc finger protein 493	*ZNF493*	0.012	1.53

FCA = Fold change absolute

-- = HUGO Gene Nomenclature Committee (HGNC) assigned unique gene name is not available

### Real-time quantitative PCR analysis

The raw data (Cp values) were read in the R software (version 2.12.0) and the non-parametric Mann-Whitney unpaired test was used for analyzing the target gene *STX11 *in reference to the housekeeping gene *ACTB*. Cp values of *ACTB *were used for normalization. Using RT-qPCR, *STX11 *was successfully amplified and confirmed to have significant differential expression (*P *= 0.036).

### *In-silico *analysis of differentially expressed genes

**Figure 2:**
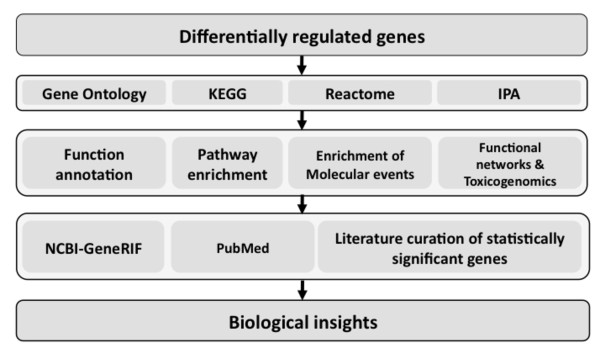
**Bioinformatics pipeline used for the biological interpretation of differentially expressed genes**.

The differentially expressed genes were analyzed using a bioinformatics pipeline to understand the functional role of the genes. A flow-chart of the multi-tiered bioinformatics approach is provided in Figure 2. Multiple resources were integrated in the pipeline to provide a cohesive view of biological functions and pathways associated with the differentially expressed genes. Results from enrichment analysis using GO terms (Tables 3 and 4), pathway analysis using KEGG pathways (Table 5) and enrichment analysis of molecular event analysis using Reactome annotations (Table 6) are provided. Interactions within the differentially regulated genes were identified using biological network analysis utilities in IPA (Tables 7 and 8). Brief description of methodology and results from various approaches are provided.

**Table 3:** Statistically significant GO terms derived from upregulated genes

Gene Ontology ID: Term	*P-value*
**Biological process** *See *Additional file 1*: Table S1* **Cellular component**	

GO:0005886: plasma membrane	0.043

**Molecular function**	

GO:0003690: double-stranded DNA binding	0.034

GO:0004725: protein tyrosine phosphatase activity	0.040

GO:0033549: MAP kinase phosphatase activity	0.040

GO:0017017: MAP kinase tyrosine/serine/threonine phosphatase activity	0.040

**Table 4:** Statistically significant GO terms derived from downregulated genes

Gene Ontology ID: Term	*P-value*
**Molecular function**	

GO:0006355: regulation of transcription, DNA-dependent	0.000

GO:0051252: regulation of RNA metabolic process	0.000

GO:0045449: regulation of transcription	0.000

GO:0006350: transcription	0.004

GO:0045941: positive regulation of transcription	0.042

GO:0010628: positive regulation of gene expression	0.045

**Biological process**	

GO:0003677: DNA binding	0.001

GO:0030528: transcription regulator activity	0.001

GO:0003700: transcription factor activity	0.002

GO:0046914: transition metal ion binding	0.040

GO:0008270: zinc ion binding	0.043

GO:0046983: protein dimerization activity	0.049

**Table 5:** KEGG Pathway enrichment analysis results

KEGG ID	Pathway Name	*P-value*
**KEGG Pathways mediated by upregulated genes**		

path:05219	Bladder cancer	0.000

path:05144	Malaria	0.000

path:04115	p53 signaling pathway	0.000

path:05140	Leishmaniasis	0.001

path:05164	Influenza A	0.001

path:05323	Rheumatoid arthritis	0.001

path:04620	Toll-like receptor signaling pathway	0.001

path:05142	Chagas disease (American trypanosomiasis)	0.001

path:04145	Phagosome	0.005

path:05200	Pathways in cancer	0.005

path:04621	NOD-like receptor signaling pathway	0.007

path:04622	RIG-I-like receptor signaling pathway	0.010

path:04010	MAPK signaling pathway	0.021

path:05146	Amoebiasis	0.022

path:00533	Glycosaminoglycan biosynthesis - keratan sulfate	0.032

path:05162	Measles	0.034

path:05160	Hepatitis C	0.034

**KEGG Pathways mediated by downregulated genes**		

path:04380	Osteoclast differentiation	0.016

path:00760	Nicotinate and nicotinamide metabolism	0.037

**Table 6:** Reactome molecular events enriched in upregulated genes

Reactome Pathway	*P-value*
Platelet Activation	0.011

Formation of Platelet plug	0.015

Exocytosis of platelet alpha granule contents	0.016

Metabolism of water-soluble vitamins and cofactors	0.017

Chemokine receptors bind chemokines	0.021

Metabolism of vitamins and cofactors	0.022

Liganded Gi-activating GPCR acts as a GEF for Gi	0.023

The Ligand:GPCR:Gi complex dissociates	0.023

Liganded Gi-activating GPCRs bind inactive heterotrimeric G-protein Gi	0.023

NFkB and MAP kinases activation mediated by TLR4 signaling repertoire	0.025

Hemostasis	0.027

MyD88-independent cascade initiated on plasma membrane	0.028

G alpha (i) signalling events	0.029

Class A/1 (Rhodopsin-like receptors)	0.030

Toll Like Receptor 10 (TLR10) Cascade	0.033

Toll Like Receptor 5 (TLR5) Cascade	0.033

MyD88 cascade initiated on plasma membrane	0.033

MyD88:Mal cascade initiated on plasma membrane	0.035

Toll Like Receptor TLR1:TLR2 Cascade	0.035

Toll Like Receptor TLR6:TLR2 Cascade	0.035

Toll Like Receptor 2 Cascade	0.035

Activated TLR4 signalling	0.040

Platelet degranulation	0.041

Toll Like Receptor 4 (TLR4) Cascade	0.042

Response to elevated platelet cytosolic Ca2+	0.046

**Table 7:** Biological networks derived using IPA network analysis

Top networks derived using upregulated genes	
**Associated functional network**	**Score**

Cell Death, Renal Necrosis/Cell Death, Cancer	39

Cardiovascular Disease, Hematological Disease, Infection Mechanism	24

Inflammatory Response, Embryonic Development, Cell-To-Cell Signaling and Interaction	21

Neurological Disease, RNA Post-Transcriptional Modification	2

Cardiovascular Disease, Genetic Disorder, Cellular Function and Maintenance	2

**Top networks derived using downregulated genes**	

Cellular Development, Cellular Assembly and Organization, Cell Cycle	13

Genetic Disorder, Ophthalmic Disease, Nervous System Development and Function	3

Embryonic Development, Gene Expression, Protein Trafficking	3

Cellular Assembly and Organization, Cell Morphology, Cellular Function and Maintenance	2

**Table 8:** Toxicity functions derived from IPA network analysis

Top Tox lists derived from upregulated genes	
**Name**	***P-value***

Renal Necrosis/Cell Death	0.000

Liver Necrosis/Cell Death	0.000

Increases Renal Proliferation	0.001

Oxidative Stress	0.001

Cardiac Necrosis/Cell Death	0.002

### GO terms associated with differentially expressed genes

GO enrichment analysis was performed using GO Fat (collection of broadest GO terms curated from GO annotations dataset) based annotations using DAVID [23,31]. The background was defined as the 'Human Genome U133 Plus 2' annotation and the differentially expressed genes from the study were input for assessing the enrichment. The upregulated and downregulated probe set identifiers were used as input and enrichment was analyzed separately and the results provided for the significantly enriched terms using Fisher's exact test using the EASE modification (*P < 0.05*) and multiple testing correction was performed using Benjamini-Hochberg FDR method. The *P-value *for each GO term reflects the enrichment in frequency of that GO term in the input entity list (differentially regulated probe set identifiers) relative to all entities in the background list (probe identifiers in Human Genome U133 Plus 2).

**Figure 3:**
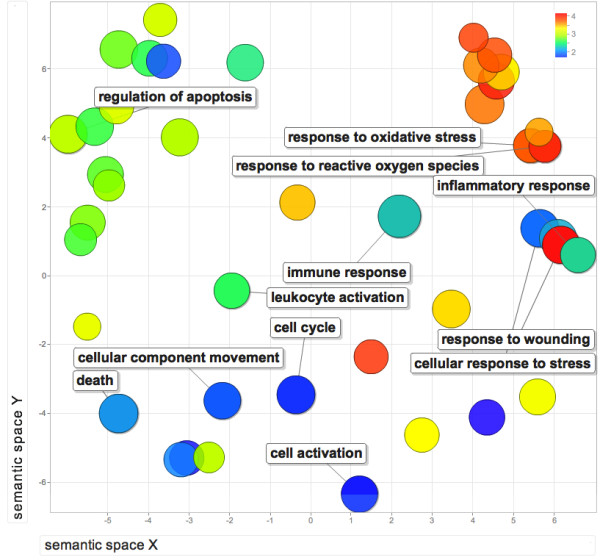
**Statistically significant GO terms (Biological Process category) derived from upregulated genes**.

Among different GO terms of upregulated genes, several enriched terms in 'biological process' (Table 3 and Additional file 1: Table S1) categories were related to molecular mechanisms associated with inflammation (inflammatory response; response to protein stimulus, response to organic substance, cytokine activity); immune response (defense response, regulation of response to external stimulus), cell death (induction of apoptosis by extracellular signals, regulation of cell proliferation, positive regulation of anti-apoptosis, regulation of apoptosis) and stress response (response to oxidative stress, response to reactive oxygen species, response to hyperoxia). Other important biological processes mediated by upregulated genes were regulation of peptidase activity, caspase activity and endopeptidase activity. A visual summary of GO identifiers associated with upregulated genes (Figure 3) were created using REVIGO [32]. Molecular functions of the upregulated genes included phosphatase activity. GO terms associated with downregulated genes were enriched for various terms related to transcriptional regulation. These results indicate that the PBMC, in the setting of PAD, differentially express genes involved in inflammation, immune response, apoptosis, molecular specific functions mediated by peptidase, caspase and stress response related pathways. Results of the GO annotation based enrichment analysis of upregulated and downregulated genes are summarized in Tables 3 and 4.

### KEGG pathways associated with differentially expressed genes

SubPathwayMiner was used to assess the statistical significance of KEGG pathways associated with differentially expressed genes. Probes were mapped to genes identifiers and gene identifiers were used as the input in the statistical analysis. The enrichment analysis revealed that 17 pathways were associated with upregulated genes and two pathways were significant in downregulated genes (*P < 0.05*) and multiple testing correction was performed using FDR. Analysis of KEGG pathway classes indicates that these pathways mediate cellular processing, signal transduction, immune system and infectious diseases. These analyses suggest that perturbations in multiple signaling and cellular mechanisms occur in PBMC in the setting of PAD. Significantly enriched pathways and corresponding *P-values *are listed in Table 5.

### Molecular events associated with PAD

Compared to classical biological pathway databases, Reactome provides biological processes as a series of molecular events and is thus a unique resource for functional interpretation of genes lists with a wide array of pathways, specific biological process and molecular events. We used the probe identifiers as the input for Reactome based enrichment analysis to find molecular events associated with differentially expressed genes using a hypergeometric test (*P < 0.05*). Pathway analysis using Reactome showed that upregulated genes were implicated in five platelet related pathways (platelet activation, formation of platelet plug, exocytosis of platelet alpha granule contents, platelet degranulation and response to elevated platelet cytosolic Ca2+). Two vitamin metabolism related events (Metabolism of water-soluble vitamins and cofactors, Metabolism of vitamins and cofactors) were also associated with upregulated genes (Table 6). Similar to KEGG pathway enrichment (Table 5), we also observed enrichment of several signal transduction events in the Reactome analysis. There was no significant enrichment of molecular events observed in the downregulated genes.

### Functional network inferred using IPA

**Figure 4:**
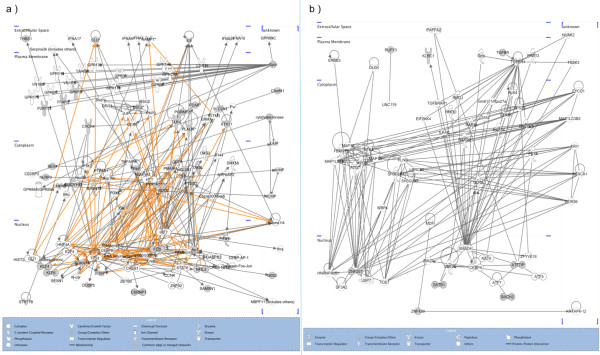
**Functional networks inferred using IPA**. a) Merged view of functional network derived from upregulated genes using IPA (Network 1: Cell Death, Renal Necrosis/Cell Death, Cancer; Network 2: Cardiovascular Disease, Hematological Disease, Infection Mechanism; Network 3: Inflammatory Response, Embryonic Development, Cell-To-Cell Signaling and Interaction Network 4: Neurological Disease, RNA Post-Transcriptional Modification; Network 5: Cardiovascular Disease, Genetic Disorder, Cellular Function and Maintenance; Network 6: Drug Metabolism, Nucleic Acid Metabolism, Small Molecular Biochemistry). b) Functional networks derived from downregulated genes using IPA (Network 1: Cellular Development, Cellular Assembly and Organization, Cell Cycle; Network 2: Genetic Disorder, Ophthalmic Disease, Nervous System Development and Function; Network 3: Embryonic Development, Gene Expression, Protein Trafficking Network 4: Cellular Assembly and Organization, Cell Morphology, Cellular Function and Maintenance).

We used IPA to understand the functionally significant biological networks and toxicogenomics associations mediated by the differentially expressed genes in the setting of PAD. IPA analysis was performed using probe identifiers as the input; the reference dataset was defined as 'Human Genome U133 Plus 2'; direct interactions only were considered for the analysis. Biological network (Table 7) and toxicity functions (Table 8) derived from IPA are provided and illustrated in Figure 4 (a) (merged view of networks derived from upregulated genes) and Figure 4 (b) (merged view of networks derived from downregulated genes). Different shapes of nodes indicate "Family" of a given gene assigned using IPA annotations. Color of node indicates the presence (grey) or absence (white) of a given gene in the study. Nodes that are not represented in the study (white nodes) were retained in the network for a context dependent view of the functional interactome. Edges shared between six different functional networks derived from upregulated genes were highlighted (Figure 4 (a)). Downregulated genes do not share any common nodes between the derived functional networks (Figure 4 (b)). It should be noted that upregulated genes interacted with several core genes (interactions are highlighted with edges colored in orange) that are present in multiple networks, where as the downregulated genes did not interact with the core genes. These results suggest that that upregulated genes identified in our study may influence multiple functional networks via interaction with the core genes. Further studies are required to understand role of these genes in the pathophysiology of PAD.

### Biocuration of differentially expressed genes

We performed in-depth biocuration of differentially regulated genes using a combination of resources. For a given differentially expressed gene we consulted General annotation under the Comments section in UniProt, RefSeq summary, GeneRIF and publications linked under "Related Articles" in PubMed section of "Bibliography" in individual Gene pages. Biocuration was performed to manually extract the role of differentially expressed pertaining to vascular diseases including PAD from previous literature reports. Curated data with functional context and role of genes in vascular diseases and associated references are presented in Additional file 1: Table S2. This approach further helped to extract functionally relevant information not captured by ontologies or annotations in automated analytical frameworks used in enrichment tools.

## Discussion

We report for the first time gene expression analysis of PBMC to identify genes differentially expressed in patients with PAD. Enrichment analysis of GO terms and pathways associated with these genes provide insights into several known and novel molecular mechanisms related to PAD. The two genes with highest fold change absolute (FCA) were: G0/G1switch 2 (*G0S2; *FCA: 3.90; ↑) BTB and CNC homology 1, basic leucine zipper transcription factor 2 (*BACH2; *FCA: 2.10; ↓). *G0S2 *is a novel target of peroxisome-proliferator-activated receptor (*PPAR) *involved in adipocyte differentiation [33,34]. *BACH2 *is a transcriptional regulator that acts as repressor or activator through the nuclear factor (erythroid-derived 2), 45 kDa (*NFE2*) binding sites [35,36]. Differentially regulated genes, summarized in Table 2 are ideal candidates for further, down-stream functional studies.

### Biological relevance of differentially expressed genes in the setting of PAD

Knowledge-based statistical analysis of differentially expressed genes provided molecular clues for the interpretation of the function or pathways associated with these genes. We used the statistically significant genes, GO terms and pathways as leads to perform in-depth literature curation. The detailed literature curation indicated that the genes identified in this study are relevant to various aspects of vascular biology and pathophysiology of PAD.

Several of the differentially regulated genes are involved in vascular pathophysiology; for example: *DNAJB6 *[37] and *DUSP1 *(atherosclerosis) [38], *NAMPT *(vascular inflammation) [39,40], *FCAR *(myocardial infarction) [41], *IL8 *(vascular remodelling) [42], *FFAR2 *(lipid metabolism) [43] and *SOD2 *(idiopathic cardiomyopathy (IDC)) [44]. Notably, several genes known to be associated with vascular disease were upregulated as discussed below.

Phosphatases are known to be associated with peripheral arterial disease [45-47]. We noted that four phosphatase genes *PTP4A1, DUSP1, DUSP5, PPP1R15A *are significantly upregulated in the PBMC of patients with PAD. Apoptosis, along with inflammation and immune response, is a key feature of vascular diseases [5,48-52]. Our study indicates genes implicated in inflammation, immune response (*FCAR, FFAR2, IL8, CFLAR, DUSP1, NAMPT*) and cell death (*G0S2, KLF6, PTP4A1, CFLAR) *are differentially expressed in PBMC of PAD patients. Oxidative-stress response is known to be associated with PAD [53-56]; we noted that several "oxidative-stress response" related functions were enriched in GO term analysis and IPA analysis. Altered metabolism of vitamins and vitamin D deficiency has been reported to be associated with PAD [57,58]. Enrichment analysis using molecular event annotations (Table 6) and GO term analysis (Table 3) indicated that vitamin metabolism related pathways are upregulated in the setting of PAD. Platelet aggregation is strongly linked to PAD [1,6,59-62] and Reactome based pathway analysis indicated that several platelet-related molecular events were associated with upregulated genes such as *PLAUR *(Table 6).

Apart from these known genes, we noted several genes not previously associated with PAD to be differentially expressed. Upregulation of validated gene *STX11 *suggests a putative role for genes associated with vesicle trafficking in the pathophysiology of PAD. Upregulation of *FFAR2 *suggests altered free fatty acid metabolism in the setting of PAD. Further investigation of differentially regulated transcription factors (for example: *C5orf41, KLF6, BACH2*), and their downstream target genes may provide additional insights into the molecular basis of PAD.

### Comparison with previous studies

Several of the differentially expressed genes identified in the current study were previously reported to be associated with various vascular biology processes. For example thrombospondin-1 (*THBS1*) [63-65], phosphatases (*DUSP1*) [45], plasminogen activator, urokinase receptor (*PLAUR*) [60], cadherins (*DSC2*) [66,67] and zinc finger proteins (*ZNF207*) [68-71] have been implicated in vascular homeostasis and pathophysiology of PAD. Prior microarray studies of PAD have also demonstrated a pattern of activation of genes involved in immune and inflammatory response [72]. Our study is designed to identify perturbed genes in PBMCs in the setting of PAD. Fu et al., [72] performed microarray analysis of atherosclerotic lesions of femoral arteries, and found that immune and inflammatory pathways were enriched in PAD cases. We replicated the following genes from Fu et. al's analysis: *CDKN1A, CXCR4, KLF4, PLAUR, SAMSN1, SOD2 *and *THBS1*. Wingrove et al., [10] performed whole-genome microarray analysis on PBMCs of 27 cases with angiographic coronary artery stenosis and 14 controls and identified 526 genes with >1.3-fold differential expression (*P *< 0.05) between cases and controls. Real time PCR in two independent cohorts (149 cases and 53 controls) for 106 genes (the 50 most significant genes and 56 additional candidate genes) confirmed that 11 genes were significantly differentially expressed between cases and controls. The differentially expressed genes that we identified in the setting of PAD did not overlap with genes found by Wingrove et al., [10] but we validated several genes differentially expressed in intermediate lesions and advanced lesions derived from femoral artery samples analyzed by Fu et al [72]. Evans et al., [73] performed microarray analysis of leg arteries and identified genes involved in inflammation and lipid uptake pathways in the setting of PAD with diabetes. Similar to observations by Evans et al., [73] we also noted that inflammation and related GO terms like immune response, apoptosis, response to stress, cell proliferation and circulation were enriched in the GO annotations of upregulated genes. Differences in methodology, sources of mRNA and the fact that PAD and CAD are distinct phenotypic manifestations of atherosclerosis may account for the varying results.

### Integrated approach for functional interpretation

We integrated four different annotation resources for functional interpretation of differentially expressed genes (Figure 2). GO annotations provided a comprehensive view of the function and processes, pathway enrichment using KEGG provided disease association of differentially expressed genes, Reactome was useful in understanding molecular events associated with genes and IPA facilitated understanding of functional networks (group of genes that share common functions) and toxicity functions. Although annotations shared several common entities, each tool provided a unique perspective of the differentially regulated genes in the setting of PAD. Further, we also employed in-depth biocuration strategies to understand the functional and pathological relevance of differentially expressed genes in the setting of vascular disease. Our integrated bioinformatics approach coupled with biocuration provided insights into the functional repertoire of differentially expressed genes.

### Strength and Limitations

A strength of this report is the application of integrative bioinformatics pipeline employed to understand the functional similarities, biological pathways, molecular events and functional networks, related to differentially expressed genes. In addition we performed in-depth literature curation to understand functional relevance of these genes. Further we validated a novel differentially regulated gene *STX11 *using qRT-PCR. Complete characterization of the genes identified in this study in the context of their relevance to PAD will require further validation and functional studies. We derived the RNA from PBMC, which may have perturbations in the cellular level due to fluctuation in cluster of differentiation 4 (CD4) count within cases and controls. Patients in our study were ascertained based on ABI (ABI ≤0.9 for cases and ABI > 1.0 for controls), additional clinical biomarkers such as T lymphocytes (T cells) and Natural killer cells (NK cells) or CD4 counts were not available.

## Conclusion

Gene expression profiling of circulating PBMC provided a global overview of differential gene expression in PAD; where 87 differentially expressed genes (47 upregulated genes and 39 downregulated genes). Integrated bioinformatics analysis of differentially regulated genes using multiple annotation tools indicated that the differentially regulated genes influence immune response, inflammation, apoptosis, various signalling pathways and various functions pertaining to vascular biology. Our whole-genome expression and bioinformatics analysis suggests that microarray based expression profiling may be useful for characterizing biomarkers for PAD. Understanding and validating groups of differentially expressed genes in the setting of PAD using PBMC can improve our understanding of the key pathophysiological mechanisms in the aetiology of PAD. Further clinical and functional studies may provide additional insights into role of the differentially expressed genes in the pathophysiology of PAD.

### Availability

Gene expression data discussed in this study was submitted to Gene Expression Omnibus (GEO) database, and can be accessed via GEO accession ID GSE27034.

## Competing interests

The authors declare that they have no competing interests.

## Authors' contributions

RM performed the experiments and contributed to the analysis. KS performed the analysis. AD contributed to the experiments. KD contributed to the analysis. KS and IJK wrote the manuscript with contributions from other authors. IJK conceived the study and provided critical input. All authors read and approved the final manuscript.

## Supplementary Material

Additional file 1 **Table S1 Statistically significant GO terms (biological process category)Supplementary**. Table S2 Functional context and biological relevance of differentially expressed genes in vascular diseases [38-40,42,43,46,68,74-146]. Click here for file
