# Supplementary material for: Gene expression profiling of peripheral blood mononuclear cells in the setting of peripheral arterial disease
Source: J Clin Bioinforma. 2012 Mar 12;2:6. doi: 10.1186/2043-9113-2-6 (PMC3381689; doi:10.1186/2043-9113-2-6)
Supplement: Additional file 1 — Table S1 Statistically significant GO terms (biological process category)Supplementary. Table S2 Functional context and biological relevance of differentially expressed genes in vascular diseases [38-40,42,43,46,68,74-146]. [file 2043-9113-2-6-S1.DOC]

**Supplementary material to *Gene Expression Profiling of Peripheral Blood Mononuclear Cells in the setting of Peripheral Arterial Disease***

Rizwan Masud^1#^, Khader Shameer^1#^, Aparna Dhar^1^, Keyue Ding^1^ and Iftikhar J. Kullo^1§^

^1^ Division of Cardiovascular Diseases, Mayo Clinic, Rochester MN 55905

^#^ Joint first authors

^§^Corresponding author

Correspondence:

Dr. Iftikhar J. Kullo

200 First Street Southwest

Rochester, MN 55905

Tel: 507-284-3423

Email: kullo.iftikhar@mayo.edu

Email addresses:

RM: drrizwan.m@gmail.com

KS: khader.shameer@mayo.edu

AD: aparnadhar86@gmail.com

KD: ding.keyue@mayo.edu

IJK: [kullo.iftikhar@mayo.edu](mailto:kullo.iftikhar@mayo.edu)

**Supplementary Material:**

Supplementary Table 1: Statistically significant GO terms (biological process category)

| Gene Ontology ID: Term | *P-value* |
| --- | --- |
| Biological process | |
| GO:0009611: response to wounding | 0.000 |
| GO:0042981: regulation of apoptosis | 0.000 |
| GO:0043067: regulation of programmed cell death | 0.000 |
| GO:0010941: regulation of cell death | 0.000 |
| GO:0006979: response to oxidative stress | 0.000 |
| GO:0010035: response to inorganic substance | 0.000 |
| GO:0006915: apoptosis | 0.000 |
| GO:0012501: programmed cell death | 0.000 |
| GO:0006955: immune response | 0.000 |
| GO:0008219: cell death | 0.000 |
| GO:0016265: death | 0.000 |
| GO:0006952: defense response | 0.000 |
| GO:0006954: inflammatory response | 0.000 |
| GO:0009991: response to extracellular stimulus | 0.001 |
| GO:0010033: response to organic substance | 0.002 |
| GO:0000302: response to reactive oxygen species | 0.002 |
| GO:0051384: response to glucocorticoid stimulus | 0.002 |
| GO:0001836: release of cytochrome c from mitochondria | 0.002 |
| GO:0043065: positive regulation of apoptosis | 0.002 |
| GO:0043068: positive regulation of programmed cell death | 0.002 |
| GO:0010942: positive regulation of cell death | 0.002 |
| GO:0031960: response to corticosteroid stimulus | 0.002 |
| GO:0048545: response to steroid hormone stimulus | 0.003 |
| GO:0040012: regulation of locomotion | 0.003 |
| GO:0006928: cell motion | 0.003 |
| GO:0034614: cellular response to reactive oxygen species | 0.004 |
| GO:0007050: cell cycle arrest | 0.004 |
| GO:0008637: apoptotic mitochondrial changes | 0.004 |
| GO:0008285: negative regulation of cell proliferation | 0.005 |
| GO:0051240: positive regulation of multicellular organismal process | 0.007 |
| GO:0034599: cellular response to oxidative stress | 0.008 |
| GO:0070482: response to oxygen levels | 0.010 |
| GO:0042127: regulation of cell proliferation | 0.011 |
| GO:0001775: cell activation | 0.012 |
| GO:0032101: regulation of response to external stimulus | 0.013 |
| GO:0007610: behavior | 0.014 |
| GO:0030334: regulation of cell migration | 0.016 |
| GO:0032103: positive regulation of response to external stimulus | 0.017 |
| GO:0031328: positive regulation of cellular biosynthetic process | 0.018 |
| GO:0033273: response to vitamin | 0.018 |
| GO:0009617: response to bacterium | 0.019 |
| GO:0009891: positive regulation of biosynthetic process | 0.019 |
| GO:0003013: circulatory system process | 0.020 |
| GO:0008015: blood circulation | 0.020 |
| GO:0009791: post-embryonic development | 0.021 |
| GO:0042060: wound healing | 0.021 |
| GO:0055093: response to hyperoxia | 0.022 |
| GO:0051270: regulation of cell motion | 0.023 |
| GO:0031667: response to nutrient levels | 0.023 |
| GO:0043066: negative regulation of apoptosis | 0.024 |
| GO:0032496: response to lipopolysaccharide | 0.025 |
| GO:0009314: response to radiation | 0.025 |
| GO:0043069: negative regulation of programmed cell death | 0.025 |
| GO:0043281: regulation of caspase activity | 0.025 |
| GO:0060548: negative regulation of cell death | 0.025 |
| GO:0006916: anti-apoptosis | 0.026 |
| GO:0052548: regulation of endopeptidase activity | 0.027 |
| GO:0007243: protein kinase cascade | 0.028 |
| GO:0009725: response to hormone stimulus | 0.028 |
| GO:0009628: response to abiotic stimulus | 0.028 |
| GO:0052547: regulation of peptidase activity | 0.030 |
| GO:0033554: cellular response to stress | 0.030 |
| GO:0002237: response to molecule of bacterial origin | 0.030 |
| GO:0042493: response to drug | 0.030 |
| GO:0007049: cell cycle | 0.031 |
| GO:0001819: positive regulation of cytokine production | 0.032 |
| GO:0044093: positive regulation of molecular function | 0.034 |
| GO:0048584: positive regulation of response to stimulus | 0.037 |
| GO:0002684: positive regulation of immune system process | 0.037 |
| GO:0009719: response to endogenous stimulus | 0.038 |
| GO:0040017: positive regulation of locomotion | 0.038 |
| GO:0008217: regulation of blood pressure | 0.040 |
| GO:0045321: leukocyte activation | 0.040 |
| GO:0009615: response to virus | 0.043 |
| GO:0051789: response to protein stimulus | 0.044 |
| GO:0050867: positive regulation of cell activation | 0.048 |
| GO:0051412: response to corticosterone stimulus | 0.049 |

Supplementary Table 2: Functional context and biological relevance of differentially expressed genes in vascular diseases

| Gene symbol | Functional or etiological context related to vascular diseases including PAD |
| --- | --- |
| **Upregulated genes** | |
| *B3GNT2* | Type II transmembrane protein involved in transferase activity, transferring glycosyl group [1] |
| *BCL2A1* | Member of Bcl-2 protein family, may involve in a protective role against endothelial cells apoptosis during inflammation [2] |
| *BEX4* | Conserved gene with high expression levels in heart, skeletal muscle and liver [3] |
| *C5orf41* | A SNP (rs251253) in *C5orf41* is associated with PR-interval [4, 5]. |
| *CDKN1A* | Differentially expressed in normal versus atherosclerotic vessels [6]. Associated with QRS duration and cardiac ventricular conduction [7] |
| *CLU* | Associated with the clearance of cellular debris and apoptosis. Protective role in atherosclerosis through its capacity to inactivate complement complex and reducing the cytotoxic effects of modified LDL [8] |
| *CMPK2* | Component of the salvage pathway for nucleotide synthesis [9] |
| *CSRNP1* | May involve in tumor suppression and apoptosis [10] |
| *CXCR4* | CXC chemokine receptor specific for stromal cell-derived factor-1. Transduces a signal by increasing intracellular calcium ion levels and enhancing MAPK1/MAPK3 activation |
| *DNAJB6* | Functional role in polyglutamine aggregation, inflammation and possible role in atherosclerosis [11]. |
| *DSC2* | Involved in interaction of plaque proteins and filaments mediated in the cell adhesion and desmosome formation. Associated with arrhythmogenic right ventricular dysplasia type 11 (ARVD11) [MIM:610476] |
| *DUSP1* | Suppresses pro-inflammatory activation at sites that are resistant to atherosclerosis [12]. |
| *DUSP5* | Plays important roles in embryonic vascular development and is mutated in vascular anomalies [13] |
| *FAM198B* |  |
| *FCAR* | Mediate immunologic response to pathogens and inflammation and involved in phagosome pathway. Carriers of 92Asn polymorphism in FCAR is associated with increased risk of myocardial infarction in CARE and increased odds of coronary heart disease in WOSCOPS study groups [14]. |
| *FFAR2* | Involved in the inflammatory response and in regulating lipid plasma level, free fatty acids and insulin [15, 16]. |
| *FOS* | Regulators of cell proliferation, differentiation, and transformation and identified as a sensitive marker of statin treatment that is dissociated from cholesterol levels [17]. |
| *G0S2* | Prominent role in apoptosis [18]. |
| *GK* | Key enzyme that regulate glycerol uptake and metabolism. Mutations in this gene are associated with glycerol kinase deficiency (GKD) [19]. |
| *GPR15* | Membrane protein involved in G-protein coupled receptor signaling pathway [20]. |
| *GUCY1B3* | Gene encodes a receptor for nitric oxide (NO) and nitrovasodilator drugs [21]. Implicated in various pathways including Vascular smooth muscle contraction (KEGG), Hemostasis (REACTOME) and Platelet homeostasis (REACTOME). |
| *HIST1H2BG* | A SNP (rs12206204-T) is reported in genome-wide association meta-analysis for total serum bilirubin levels [22]. |
| *IFIH1* | Involved in alteration of alteration of RNA secondary structure [23]. Gene is associated with Diabetes mellitus, insulin-dependent, 19 (IDDM19) (MIM: 610155) and associated with type 1 diabetes [24]. |
| *IL8* | Enhance vascular remodelling tumor formation and metastasis, with evidence suggesting that it inhibits apoptosis of cells that express receptors for this cytokine [25] . |
| *KLF4* | Transcription factor implicated in fat cell differentiation, Diabetes pathways (REACTOME) and Transcriptional Regulation of White Adipocyte Differentiation (REACTOME) pathways. Influence the expression of Erk5 that elicits an overall protective phenotype characterized by increased apoptosis resistance and a decreased angiogenic, migratory, and inflammatory potential [26]. |
| *KLF6* | A transcriptional activator of insulin-like growth factor I receptor [27] with functional implication as a tumor suppressor [28]. |
| *NAMPT* | Plays a crucial role in the promotion of vascular smooth muscle cell maturation, inhibition of neutrophil apoptosis, pathogenesis of type-2 diabetes, regulates insulin activity that includes lowering blood glucose and improving insulin resistivity. *NAMPT* is shown to be contributor of ‘vascular inflammation’, an atherothrombotic disease linked to metabolic disorders [29-31] |
| *NFIL3* | Transcription factor with a prominent role as cardiac survival factor and essential for embryonic heart development [32]. |
| *NLRP3* | Intronic SNPs are associated with circulating fibrinogen levels [33] and C-reactive protein levels [34] |
| *OASL* | Higher expression levels are associated with systemic lupus erythematosus. Associated with Type 2 diabetes [35] and N-glycan levels [36]. |
| *P2RY12* | G-protein coupled receptor associated with platelet aggregation and a drug target for the treatment of thromboembolisms and other clotting disorders [37] . *P2RY12* mediate platelet-independent responses, specifically under conditions of enhanced thrombin formation, such as local vessel injury and atherosclerotic plaque rupture [38]. |
| *PGRMC1* | Membrane bound progesterone steroid receptor and implicated in prevention of apoptosis [39]. |
| *PLAUR* | Implicated in inflammation, promote plasmin formation, and regulate plasminogen activation and localized degradation of the extracellular matrix [40, 41]. |
| *PMAIP1* | Promotes activation of caspases and apoptosis [42]. |
| *PPP1R15A* | Associated with functions in cardiac tissue in response to ischaemia [43] |
| *PTGS2* | Inducible isozyme implicated in the prostanoid biosynthesis involved in inflammation and mitogenesis [44]. Associated with vasculopathies including atherothrombosis [45]. |
| *PTP4A1* | Affect cell growth and tumour development by the downregulation of p53 using a negative feedback mechanism [46] |
| *SAMSN1* | Involved in B cell activation and differentiation, Metabolic disorders, Cirrohsis, Steatosis, and steatohepatitis [47, 48] |
| *SLC2A14* | A transmembrane protein, facilitate glucose transport and member of glucose transporter (GLUT) family [49] |
| *SOD2* | Associated with idiopathic cardiomyopathy (IDC) [50] |
| *STK17B* | Involved in the regulation of apoptosis |
| *STX11* | Cellular trafficking including targeting and fusion of intracellular transport vesicles [51] |
| *TAGAP* | Function as a Rho GTPase-activating protein and associated with Crohn’s disease [52], rheumatoid arthritis [53], type 1 diabetes and coeliac disease [54] |
| *THBS1* | Adhesive glycoprotein that mediates cell-to-cell and cell-to-matrix interactions. Interact with fibrinogen, fibronectin, laminin, type V collagen and integrins alpha-V/beta-1. Implicated in platelet aggregation, angiogenesis, and tumorigenesis [55, 56]. |
| *TLR4* | Member of toll-like receptor (TLR) family which plays a fundamental role in pathogen recognition and activation of innate immunity. Play an important role in vascular inflammation [57, 58]. |
| *TPM1* | Actin-binding proteins involved in the contractile system of striated and smooth muscles and the cytoskeleton of non-muscle cells. Associated with type 3 familial hypertrophic cardiomyopathy (MIM: 115196) and dilated cardiomyopathy (MIM: 611878) [59] |
| *TRBC1* | Single-pass membrane protein with a Ig-like (immunoglobulin-like) domain (UniProt). Part of T cell receptor signaling pathway (Reactome) |
| **Downregulated genes** | |
| *AFF1* | An intergenic variant proximal to *AFF1* is associated with decrease in triglycerides level [60]. |
| *ANKRD46* | An intergenic SNP is associated with increase in atrioventricular conduction [61]. |
| *ATF7IP* | Transcriptional coactivator associated with heterochromatin [62]. |
| *BACH2* | Transcriptional regulator that acts as repressor or activator through the nuclear factor (erythroid-derived 2), 45kDa (*NFE2*) binding. Associated with Crohn’s disease [52] and juvenile idiopathic arthritis [60]. |
| *BLNK* | Plays a critical role in B cell development and act as a linker that bridge B cell receptors and several signaling pathways [63]. |
| *BMP2KL* | This gene encodes a putative BMP-2-inducible kinase-like protein and may involved in protein serine/threonine kinase activity (UniProt). |
| *C14orf43* | Part of a mitotic deacetylase complex (MIDAC) [64]. |
| *C5orf28* | Conserved membrane protein with no known prominent biochemical function or known interaction partner (UniProt). |
| *C7orf44* | Putative membrane protein with no known prominent biochemical function or known interaction partner (UniProt). |
| *CASD1* | Involved in O-acetylation of alpha2-8-linked sialic acids, which are important sugars at the reducing end of glycoproteins and glycolipids [65]. |
| *DENND1B* | Member of the connecdenn family, involved in activation of clathrin machinery [66]. |
| *DIS3L2* | This gene encodes a putative exonuclease and may involve in RNA binding (UniProt). |
| *EBF1* | Transcription factor that may induce adipogenesis in induce adipogenesis of NIH-3T3 cells [67] |
| *FAM129C* | Encodes a member of Niban family and specifically expressed in B-lymphocytes [68]. |
| *FCRL2* | Member of immunoglobulin receptor superfamily with immunomodulatory potential [69]. |
| *FTX* | Non-coding RNA which affects expression of *XIST* [70] |
| *GPR141* | Member of the rhodopsin family of G protein-coupled receptors (GPRs) and involved in G-protein coupled receptor signaling pathway [71]. |
| *KLRC1* | Transmembrane proteins preferentially expressed in NK cells that regulate specific humoral and cell-mediated immunity . |
| *MALAT1* | Long non-coding RNA known to influence expression of motility-related genes [72] |
| *METT5D1* | Putative S-adenosyl-L-methionine-dependent methyltransferase (UniProt) |
| *MIR181A2HG* | Human miRNA predicted by computational methods using conservation with mouse and *Fugu rubripes* sequences (miRBase) |
| *NR3C2* | Encodes a mineralocorticoid receptor, which mediates aldosterone actions on salt and water balance within restricted target cells. May have functional role in vascular dysfunction [73]. |
| *NT5C3* | Member of nucleotidase gene family, which encodes the type I pyrimidine 5'-nucleotidase (P5N) isozyme [74]. |
| *PPP1R7* | Putative regulatory subunit of protein phosphatase 1 and may contribute to # tumorigenesis and metastatic potential [75] |
| *RGPD1* | Protein encodes a GRIP domain, 2 Tetratricopeptide-like helical domains and 2 Ran binding protein 1 (UniProt). |
| *RUFY3* | It encodes a developmental protein with RUN domains involved in Ras-like GTPase signaling [76]. |
| *SATB1* | Encodes a matrix protein which binds nuclear matrix and scaffold-associating DNAs through a unique nuclear architecture to regulate chromatin structure and gene expression [77]. |
| *TGFBR1* | Transmembrane serine/threonine protein kinase implicated in artery morphogenesis and heart development [78]. |
| *WWOX* | Tumor suppressor protein with 2 WW domains and a short-chain dehydrogenase/reductase domain (SRD). May involved in tumor necrosis factor-alpha-induced apoptosis [79]. |
| *ZCCHC7* | Component of RNA degradation and TRAMP complex pathways (KEGG) |
| *ZNF207* | Transcription factor with C2H2-type zinc finger domains [80]. |
| *ZNF439* | Transcription factor with 11 C2H2-type zinc finger domains and 1 KRAB domain (UniProt) |
| *ZNF479* | Transcription factor with 12 C2H2-type zinc finger domains and 1 KRAB domain (UniProt) |
| *ZNF493* | Transcription factor with 22 C2H2-type zinc finger domains (UniProt) |

**References:**

1. Seko A, Yamashita K: **Activation of beta1,3-N-acetylglucosaminyltransferase-2 (beta3Gn-T2) by beta3Gn-T8. Possible involvement of beta3Gn-T8 in increasing poly-N-acetyllactosamine chains in differentiated HL-60 cells**. *J Biol Chem* 2008, **283**(48):33094-33100.

2. Karsan A, Yee E, Kaushansky K, Harlan JM: **Cloning of human Bcl-2 homologue: inflammatory cytokines induce human A1 in cultured endothelial cells**. *Blood* 1996, **87**(8):3089-3096.

3. Alvarez E, Zhou W, Witta SE, Freed CR: **Characterization of the Bex gene family in humans, mice, and rats**. *Gene* 2005, **357**(1):18-28.

4. Pfeufer A, van Noord C, Marciante KD, Arking DE, Larson MG, Smith AV, Tarasov KV, Muller M, Sotoodehnia N, Sinner MF *et al*: **Genome-wide association study of PR interval**. *Nat Genet* 2010, **42**(2):153-159.

5. Audas TE, Li Y, Liang G, Lu R: **A novel protein, Luman/CREB3 recruitment factor, inhibits Luman activation of the unfolded protein response**. *Mol Cell Biol* 2008, **28**(12):3952-3966.

6. Rodriguez I, Coto E, Reguero JR, Gonzalez P, Andres V, Lozano I, Martin M, Alvarez V, Moris C: **Role of the CDKN1A/p21, CDKN1C/p57, and CDKN2A/p16 genes in the risk of atherosclerosis and myocardial infarction**. *Cell Cycle* 2007, **6**(5):620-625.

7. Sotoodehnia N, Isaacs A, de Bakker PI, Dorr M, Newton-Cheh C, Nolte IM, van der Harst P, Muller M, Eijgelsheim M, Alonso A *et al*: **Common variants in 22 loci are associated with QRS duration and cardiac ventricular conduction**. *Nat Genet* 2010, **42**(12):1068-1076.

8. Schwarz M, Spath L, Lux CA, Paprotka K, Torzewski M, Dersch K, Koch-Brandt C, Husmann M, Bhakdi S: **Potential protective role of apoprotein J (clusterin) in atherogenesis: binding to enzymatically modified low-density lipoprotein reduces fatty acid-mediated cytotoxicity**. *Thromb Haemost* 2008, **100**(1):110-118.

9. Xu Y, Johansson M, Karlsson A: **Human UMP-CMP kinase 2, a novel nucleoside monophosphate kinase localized in mitochondria**. *J Biol Chem* 2008, **283**(3):1563-1571.

10. Ishiguro H, Tsunoda T, Tanaka T, Fujii Y, Nakamura Y, Furukawa Y: **Identification of AXUD1, a novel human gene induced by AXIN1 and its reduced expression in human carcinomas of the lung, liver, colon and kidney**. *Oncogene* 2001, **20**(36):5062-5066.

11. Gargalovic PS, Imura M, Zhang B, Gharavi NM, Clark MJ, Pagnon J, Yang WP, He A, Truong A, Patel S *et al*: **Identification of inflammatory gene modules based on variations of human endothelial cell responses to oxidized lipids**. *Proceedings of the National Academy of Sciences of the United States of America* 2006, **103**(34):12741-12746.

12. Zakkar M, Chaudhury H, Sandvik G, Enesa K, Luong le A, Cuhlmann S, Mason JC, Krams R, Clark AR, Haskard DO *et al*: **Increased endothelial mitogen-activated protein kinase phosphatase-1 expression suppresses proinflammatory activation at sites that are resistant to atherosclerosis**. *Circ Res* 2008, **103**(7):726-732.

13. Pramanik K, Chun CZ, Garnaas MK, Samant GV, Li K, Horswill MA, North PE, Ramchandran R: **Dusp-5 and Snrk-1 coordinately function during vascular development and disease**. *Blood* 2009, **113**(5):1184-1191.

14. Ferreira VP, Pangburn MK, Cortes C: **Complement control protein factor H: the good, the bad, and the inadequate**. *Mol Immunol* 2010, **47**(13):2187-2197.

15. Lee T, Schwandner R, Swaminath G, Weiszmann J, Cardozo M, Greenberg J, Jaeckel P, Ge H, Wang Y, Jiao X *et al*: **Identification and functional characterization of allosteric agonists for the G protein-coupled receptor FFA2**. *Mol Pharmacol* 2008, **74**(6):1599-1609.

16. Swaminath G: **Fatty acid binding receptors and their physiological role in type 2 diabetes**. *Arch Pharm (Weinheim)* 2008, **341**(12):753-761.

17. Kang JG, Sung HJ, Jawed SI, Brenneman CL, Rao YN, Sher S, Facio FM, Biesecker LG, Quyyumi AA, Sachdev V *et al*: **FOS expression in blood as a LDL-independent marker of statin treatment**. *Atherosclerosis* 2010, **212**(2):567-570.

18. Welch C, Santra MK, El-Assaad W, Zhu X, Huber WE, Keys RA, Teodoro JG, Green MR: **Identification of a protein, G0S2, that lacks Bcl-2 homology domains and interacts with and antagonizes Bcl-2**. *Cancer Res* 2009, **69**(17):6782-6789.

19. Hellerud C, Burlina A, Gabelli C, Ellis JR, Nyholm PG, Lindstedt S: **Glycerol metabolism and the determination of triglycerides--clinical, biochemical and molecular findings in six subjects**. *Clin Chem Lab Med* 2003, **41**(1):46-55.

20. Heiber M, Marchese A, Nguyen T, Heng HH, George SR, O'Dowd BF: **A novel human gene encoding a G-protein-coupled receptor (GPR15) is located on chromosome 3**. *Genomics* 1996, **32**(3):462-465.

21. Brandes RP, Kim D, Schmitz-Winnenthal FH, Amidi M, Godecke A, Mulsch A, Busse R: **Increased nitrovasodilator sensitivity in endothelial nitric oxide synthase knockout mice: role of soluble guanylyl cyclase**. *Hypertension* 2000, **35**(1 Pt 2):231-236.

22. Johnson AD, Kavousi M, Smith AV, Chen MH, Dehghan A, Aspelund T, Lin JP, van Duijn CM, Harris TB, Cupples LA *et al*: **Genome-wide association meta-analysis for total serum bilirubin levels**. *Human molecular genetics* 2009, **18**(14):2700-2710.

23. Mibayashi M, Martinez-Sobrido L, Loo YM, Cardenas WB, Gale M, Jr., Garcia-Sastre A: **Inhibition of retinoic acid-inducible gene I-mediated induction of beta interferon by the NS1 protein of influenza A virus**. *J Virol* 2007, **81**(2):514-524.

24. Barrett JC, Clayton DG, Concannon P, Akolkar B, Cooper JD, Erlich HA, Julier C, Morahan G, Nerup J, Nierras C *et al*: **Genome-wide association study and meta-analysis find that over 40 loci affect risk of type 1 diabetes**. *Nat Genet* 2009, **41**(6):703-707.

25. Li A, Dubey S, Varney ML, Dave BJ, Singh RK: **IL-8 directly enhanced endothelial cell survival, proliferation, and matrix metalloproteinases production and regulated angiogenesis**. *J Immunol* 2003, **170**(6):3369-3376.

26. Ohnesorge N, Viemann D, Schmidt N, Czymai T, Spiering D, Schmolke M, Ludwig S, Roth J, Goebeler M, Schmidt M: **Erk5 activation elicits a vasoprotective endothelial phenotype via induction of Kruppel-like factor 4 (KLF4)**. *J Biol Chem* 2010, **285**(34):26199-26210.

27. Rubinstein M, Idelman G, Plymate SR, Narla G, Friedman SL, Werner H: **Transcriptional activation of the insulin-like growth factor I receptor gene by the Kruppel-like factor 6 (KLF6) tumor suppressor protein: potential interactions between KLF6 and p53**. *Endocrinology* 2004, **145**(8):3769-3777.

28. Andreoli V, Gehrau RC, Bocco JL: **Biology of Kruppel-like factor 6 transcriptional regulator in cell life and death**. *IUBMB Life* 2010, **62**(12):896-905.

29. Kadoglou NP, Sailer N, Moumtzouoglou A, Kapelouzou A, Tsanikidis H, Vitta I, Karkos C, Karayannacos PE, Gerasimidis T, Liapis CD: **Visfatin (nampt) and ghrelin as novel markers of carotid atherosclerosis in patients with type 2 diabetes**. *Exp Clin Endocrinol Diabetes* 2010, **118**(2):75-80.

30. Laudes M, Oberhauser F, Schulte DM, Freude S, Bilkovski R, Mauer J, Rappl G, Abken H, Hahn M, Schulz O *et al*: **Visfatin/PBEF/Nampt and resistin expressions in circulating blood monocytes are differentially related to obesity and type 2 diabetes in humans**. *Horm Metab Res* 2010, **42**(4):268-273.

31. Romacho T, Azcutia V, Vazquez-Bella M, Matesanz N, Cercas E, Nevado J, Carraro R, Rodriguez-Manas L, Sanchez-Ferrer CF, Peiro C: **Extracellular PBEF/NAMPT/visfatin activates pro-inflammatory signalling in human vascular smooth muscle cells through nicotinamide phosphoribosyltransferase activity**. *Diabetologia* 2009, **52**(11):2455-2463.

32. Weng YJ, Hsieh DJ, Kuo WW, Lai TY, Hsu HH, Tsai CH, Tsai FJ, Lin DY, Lin JA, Huang CY *et al*: **E4BP4 is a cardiac survival factor and essential for embryonic heart development**. *Mol Cell Biochem* 2010, **340**(1-2):187-194.

33. Dehghan A, Yang Q, Peters A, Basu S, Bis JC, Rudnicka AR, Kavousi M, Chen MH, Baumert J, Lowe GD *et al*: **Association of novel genetic Loci with circulating fibrinogen levels: a genome-wide association study in 6 population-based cohorts**. *Circ Cardiovasc Genet* 2009, **2**(2):125-133.

34. Dehghan A, Dupuis J, Barbalic M, Bis JC, Eiriksdottir G, Lu C, Pellikka N, Wallaschofski H, Kettunen J, Henneman P *et al*: **Meta-analysis of genome-wide association studies in >80 000 subjects identifies multiple loci for C-reactive protein levels**. *Circulation* 2011, **123**(7):731-738.

35. Voight BF, Scott LJ, Steinthorsdottir V, Morris AP, Dina C, Welch RP, Zeggini E, Huth C, Aulchenko YS, Thorleifsson G *et al*: **Twelve type 2 diabetes susceptibility loci identified through large-scale association analysis**. *Nat Genet* 2010, **42**(7):579-589.

36. Lauc G, Essafi A, Huffman JE, Hayward C, Knezevic A, Kattla JJ, Polasek O, Gornik O, Vitart V, Abrahams JL *et al*: **Genomics meets glycomics-the first GWAS study of human N-Glycome identifies HNF1alpha as a master regulator of plasma protein fucosylation**. *PLoS Genet* 2010, **6**(12):e1001256.

37. Dorsam RT, Kunapuli SP: **Central role of the P2Y12 receptor in platelet activation**. *J Clin Invest* 2004, **113**(3):340-345.

38. Rauch BH, Rosenkranz AC, Ermler S, Bohm A, Driessen J, Fischer JW, Sugidachi A, Jakubowski JA, Schror K: **Regulation of functionally active P2Y12 ADP receptors by thrombin in human smooth muscle cells and the presence of P2Y12 in carotid artery lesions**. *Arteriosclerosis, thrombosis, and vascular biology* 2010, **30**(12):2434-2442.

39. Peluso JJ, Liu X, Gawkowska A, Johnston-MacAnanny E: **Progesterone activates a progesterone receptor membrane component 1-dependent mechanism that promotes human granulosa/luteal cell survival but not progesterone secretion**. *J Clin Endocrinol Metab* 2009, **94**(7):2644-2649.

40. Alfano D, Franco P, Vocca I, Gambi N, Pisa V, Mancini A, Caputi M, Carriero MV, Iaccarino I, Stoppelli MP: **The urokinase plasminogen activator and its receptor: role in cell growth and apoptosis**. *Thromb Haemost* 2005, **93**(2):205-211.

41. Chavakis T, Kanse SM, May AE, Preissner KT: **Haemostatic factors occupy new territory: the role of the urokinase receptor system and kininogen in inflammation**. *Biochem Soc Trans* 2002, **30**(2):168-173.

42. Oda E, Ohki R, Murasawa H, Nemoto J, Shibue T, Yamashita T, Tokino T, Taniguchi T, Tanaka N: **Noxa, a BH3-only member of the Bcl-2 family and candidate mediator of p53-induced apoptosis**. *Science* 2000, **288**(5468):1053-1058.

43. Morton E, Macrae IM, McCabe C, Brown SM, White F: **Identification of the growth arrest and DNA damage protein GADD34 in the normal human heart and demonstration of alterations in expression following myocardial ischaemia**. *Int J Cardiol* 2006, **107**(1):126-129.

44. Cipollone F, Fazia ML: **COX-2 and atherosclerosis**. *J Cardiovasc Pharmacol* 2006, **47 Suppl 1**:S26-36.

45. Cipollone F, Toniato E, Martinotti S, Mezzetti A: **Genetic and molecular determinants of atherosclerotic plaque instability**. *Curr Vasc Pharmacol* 2010, **8**(4):545-552.

46. Min SH, Kim DM, Heo YS, Kim YI, Kim HM, Kim J, Han YM, Kim IC, Yoo OJ: **New p53 target, phosphatase of regenerating liver 1 (PRL-1) downregulates p53**. *Oncogene* 2009, **28**(4):545-554.

47. Claudio JO, Zhu YX, Benn SJ, Shukla AH, McGlade CJ, Falcioni N, Stewart AK: **HACS1 encodes a novel SH3-SAM adaptor protein differentially expressed in normal and malignant hematopoietic cells**. *Oncogene* 2001, **20**(38):5373-5377.

48. Zhu YX, Benn S, Li ZH, Wei E, Masih-Khan E, Trieu Y, Bali M, McGlade CJ, Claudio JO, Stewart AK: **The SH3-SAM adaptor HACS1 is up-regulated in B cell activation signaling cascades**. *J Exp Med* 2004, **200**(6):737-747.

49. Joost HG, Bell GI, Best JD, Birnbaum MJ, Charron MJ, Chen YT, Doege H, James DE, Lodish HF, Moley KH *et al*: **Nomenclature of the GLUT/SLC2A family of sugar/polyol transport facilitators**. *Am J Physiol Endocrinol Metab* 2002, **282**(4):E974-976.

50. Charniot JC, Sutton A, Bonnefont-Rousselot D, Cosson C, Khani-Bittar R, Giral P, Charnaux N, Albertini JP: **Manganese superoxide dismutase dimorphism relationship with severity and prognosis in cardiogenic shock due to dilated cardiomyopathy**. *Free Radic Res* 2011, **45**(4):379-388.

51. Valdez AC, Cabaniols JP, Brown MJ, Roche PA: **Syntaxin 11 is associated with SNAP-23 on late endosomes and the trans-Golgi network**. *J Cell Sci* 1999, **112 ( Pt 6)**:845-854.

52. Franke A, McGovern DP, Barrett JC, Wang K, Radford-Smith GL, Ahmad T, Lees CW, Balschun T, Lee J, Roberts R *et al*: **Genome-wide meta-analysis increases to 71 the number of confirmed Crohn's disease susceptibility loci**. *Nat Genet* 2010, **42**(12):1118-1125.

53. Chen R, Stahl EA, Kurreeman FA, Gregersen PK, Siminovitch KA, Worthington J, Padyukov L, Raychaudhuri S, Plenge RM: **Fine mapping the TAGAP risk locus in rheumatoid arthritis**. *Genes Immun* 2011, **12**(4):314-318.

54. Eyre S, Hinks A, Bowes J, Flynn E, Martin P, Wilson AG, Morgan AW, Emery P, Steer S, Hocking LJ *et al*: **Overlapping genetic susceptibility variants between three autoimmune disorders: rheumatoid arthritis, type 1 diabetes and coeliac disease**. *Arthritis Res Ther* 2010, **12**(5):R175.

55. Esemuede N, Lee T, Pierre-Paul D, Sumpio BE, Gahtan V: **The role of thrombospondin-1 in human disease**. *J Surg Res* 2004, **122**(1):135-142.

56. Sargiannidou I, Qiu C, Tuszynski GP: **Mechanisms of thrombospondin-1-mediated metastasis and angiogenesis**. *Semin Thromb Hemost* 2004, **30**(1):127-136.

57. Stoll LL, Denning GM, Weintraub NL: **Endotoxin, TLR4 signaling and vascular inflammation: potential therapeutic targets in cardiovascular disease**. *Curr Pharm Des* 2006, **12**(32):4229-4245.

58. An H, Qian C, Cao X: **Regulation of Toll-like receptor signaling in the innate immunity**. *Sci China Life Sci* 2010, **53**(1):34-43.

59. Perry SV: **Vertebrate tropomyosin: distribution, properties and function**. *J Muscle Res Cell Motil* 2001, **22**(1):5-49.

60. Waterworth DM, Ricketts SL, Song K, Chen L, Zhao JH, Ripatti S, Aulchenko YS, Zhang W, Yuan X, Lim N *et al*: **Genetic variants influencing circulating lipid levels and risk of coronary artery disease**. *Arteriosclerosis, thrombosis, and vascular biology* 2010, **30**(11):2264-2276.

61. Denny JC, Ritchie MD, Crawford DC, Schildcrout JS, Ramirez AH, Pulley JM, Basford MA, Masys DR, Haines JL, Roden DM: **Identification of genomic predictors of atrioventricular conduction: using electronic medical records as a tool for genome science**. *Circulation* 2010, **122**(20):2016-2021.

62. Ichimura T, Watanabe S, Sakamoto Y, Aoto T, Fujita N, Nakao M: **Transcriptional repression and heterochromatin formation by MBD1 and MCAF/AM family proteins**. *J Biol Chem* 2005, **280**(14):13928-13935.

63. Fu C, Turck CW, Kurosaki T, Chan AC: **BLNK: a central linker protein in B cell activation**. *Immunity* 1998, **9**(1):93-103.

64. Bantscheff M, Hopf C, Savitski MM, Dittmann A, Grandi P, Michon AM, Schlegl J, Abraham Y, Becher I, Bergamini G *et al*: **Chemoproteomics profiling of HDAC inhibitors reveals selective targeting of HDAC complexes**. *Nat Biotechnol* 2011, **29**(3):255-265.

65. Arming S, Wipfler D, Mayr J, Merling A, Vilas U, Schauer R, Schwartz-Albiez R, Vlasak R: **The human Cas1 protein: a sialic acid-specific O-acetyltransferase?** *Glycobiology* 2011, **21**(5):553-564.

66. Marat AL, McPherson PS: **The connecdenn family, Rab35 guanine nucleotide exchange factors interfacing with the clathrin machinery**. *J Biol Chem* 2010, **285**(14):10627-10637.

67. Akerblad P, Mansson R, Lagergren A, Westerlund S, Basta B, Lind U, Thelin A, Gisler R, Liberg D, Nelander S *et al*: **Gene expression analysis suggests that EBF-1 and PPARgamma2 induce adipogenesis of NIH-3T3 cells with similar efficiency and kinetics**. *Physiological genomics* 2005, **23**(2):206-216.

68. Boyd RS, Adam PJ, Patel S, Loader JA, Berry J, Redpath NT, Poyser HR, Fletcher GC, Burgess NA, Stamps AC *et al*: **Proteomic analysis of the cell-surface membrane in chronic lymphocytic leukemia: identification of two novel proteins, BCNP1 and MIG2B**. *Leukemia* 2003, **17**(8):1605-1612.

69. Davis RS, Wang YH, Kubagawa H, Cooper MD: **Identification of a family of Fc receptor homologs with preferential B cell expression**. *Proceedings of the National Academy of Sciences of the United States of America* 2001, **98**(17):9772-9777.

70. Chureau C, Chantalat S, Romito A, Galvani A, Duret L, Avner P, Rougeulle C: **Ftx is a non-coding RNA which affects Xist expression and chromatin structure within the X-inactivation center region**. *Hum Mol Genet* 2011, **20**(4):705-718.

71. Fredriksson R, Hoglund PJ, Gloriam DE, Lagerstrom MC, Schioth HB: **Seven evolutionarily conserved human rhodopsin G protein-coupled receptors lacking close relatives**. *FEBS Lett* 2003, **554**(3):381-388.

72. Tano K, Mizuno R, Okada T, Rakwal R, Shibato J, Masuo Y, Ijiri K, Akimitsu N: **MALAT-1 enhances cell motility of lung adenocarcinoma cells by influencing the expression of motility-related genes**. *FEBS Lett* 2010, **584**(22):4575-4580.

73. Favre J, Gao J, Zhang AD, Remy-Jouet I, Ouvrard-Pascaud A, Dautreaux B, Escoubet B, Thuillez C, Jaisser F, Richard V: **Coronary endothelial dysfunction after cardiomyocyte-specific mineralocorticoid receptor overexpression**. *Am J Physiol Heart Circ Physiol* 2011, **300**(6):H2035-2043.

74. Aksoy P, Zhu MJ, Kalari KR, Moon I, Pelleymounter LL, Eckloff BW, Wieben ED, Yee VC, Weinshilboum RM, Wang L: **Cytosolic 5'-nucleotidase III (NT5C3): gene sequence variation and functional genomics**. *Pharmacogenet Genomics* 2009, **19**(8):567-576.

75. Jiang Y, Scott KL, Kwak SJ, Chen R, Mardon G: **Sds22/PP1 links epithelial integrity and tumor suppression via regulation of myosin II and JNK signaling**. *Oncogene* 2011, **30**(29):3248-3260.

76. Callebaut I, de Gunzburg J, Goud B, Mornon JP: **RUN domains: a new family of domains involved in Ras-like GTPase signaling**. *Trends Biochem Sci* 2001, **26**(2):79-83.

77. Wen J, Huang S, Rogers H, Dickinson LA, Kohwi-Shigematsu T, Noguchi CT: **SATB1 family protein expressed during early erythroid differentiation modifies globin gene expression**. *Blood* 2005, **105**(8):3330-3339.

78. Massague J: **TGF-beta signal transduction**. *Annu Rev Biochem* 1998, **67**:753-791.

79. Bednarek AK, Keck-Waggoner CL, Daniel RL, Laflin KJ, Bergsagel PL, Kiguchi K, Brenner AJ, Aldaz CM: **WWOX, the FRA16D gene, behaves as a suppressor of tumor growth**. *Cancer Res* 2001, **61**(22):8068-8073.

80. Pahl PM, Hodges YK, Meltesen L, Perryman MB, Horwitz KB, Horwitz LD: **ZNF207, a ubiquitously expressed zinc finger gene on chromosome 6p21.3**. *Genomics* 1998, **53**(3):410-412.
